
JOURNAL INFORMATION
==============================
NLM Title Abbreviation: South Med Rec
NLM Journal ID: 0027317

The Southern Medical Record

Publisher: Biomedical Journal Digitization Project

ARTICLE INFORMATION
==============================
PMCID: PMC9094927
PMID: 36024227
Article version: 1
Subjects: Editorial

Baltimore College of Physicians and Surgeons

Opie Thomas M. D. Dean, N. W. Cor. Colvert and Saratoga St., Baltimore, Md.

Print publication date: 1889 Aug
Volume: 19
Issue: 8
First page: 318
Last page: 318
License: This work is free of known copyright restrictions. For more information, please see PMC Back Issue Digitization.
License URL: https://creativecommons.org/publicdomain/mark/1.0/

==============================

COLLEGE OF PHYSICIANS AND SURGEONS,

OF

BALTIMORE, MAINE.

The preliminary course will begin September 15th, 1889. The Regular Session will open Oct. 1st, 1889, and close about March 15th, 1890.

For catalogue containing full particulars address

THOMAS OPIE, M. D., DEAN,

N. W. Cor. Colvert and Saratoga St., Baltimore, Md.

August No. 2 times.

---

### SPECIAL NOTES.

**To Medical Students.**—The Southern Medical College, Atlanta, Georgia, is now regarded as among the very best Institutions in the United States. The College building is commodious and well arranged. The Chairs are well and ably filled. The curriculum is complete. The Dissecting-room is large and well-fitted up for the purpose, and every facility exists for imparting a thorough medical education. The Annual Catalogue is now being distributed. Parties who desire a Catalogue should address,

W. P. NICOLSON, M. D., DEAN, Atlanta, Ga.

For information in regard to the Dental Department, address,

WM. CRENSHAW, D. D. S.,

24½ Whitehall St., Atlanta, Ga.

"Am prescribing TONGALINE with satisfactory results. For the indefinite aches and pains of nervous patients it is *superior to any other anodyne*. For Nervous Headache or Muscular Rheumatism it is almost a specific.

**Bromidia, Papine and Iodia,** as prepared by Battle & Co., St. Louis, are all valuable preparations. Examine the advertisement of this House as found in this Journal.

**Wm. R. Warner & Co.,** the well known wholesale druggists and manufacturing chemists of Philadelphia, have attained a world wide reputation. They have obtained no less than ten World's fair medals. The great New Orleans exposition gave them a gold medal for quick solubility and uniformity of their sugar coated preparations, and the great Paris exposition awarded them the Vermeel medal for superiority.
